# Evaluation of the attitudes of dental students about interprofessional learning using the RIPLS questionnaire

**DOI:** 10.1186/s12909-025-07505-z

**Published:** 2025-07-21

**Authors:** Saber Mohammadi, Sara Pourshahidi, Mohammad javad Kharazifard, Kimia HafeziMotlagh, Arghavan Tonkaboni

**Affiliations:** 1 School of Dentistry, Tehran University of Medical Sciences, Tehran, Iran; 2 Oral Medicine Department, School of Dentistry, Tehran University of Medical Sciences, Tehran, Iran; 3 Epidemiologist, Dental Research Center, Dentistry Research Institute, Tehran University of Medical Sciences, Tehran, Iran; 4 Medical-Surgical Oral Pathology Research Group, University of Santiago de Compostela, Santiago de Compostela, Spain

**Keywords:** Attitude, Dental students, Interprofessional education, Knowledge, Surveys and questionnaires, Reproducibility, Education, Interprofessional

## Abstract

**Objectives:**

This study aimed to assess the attitude of Iranian dental students towards interprofessional education (IPE).

**Materials and methods:**

This cross-sectional study was conducted on a diverse group of 235 third-, fourth—, fifth–, and sixth-year dental students from Tehran University of Medical Sciences in 2020–2021. The Readiness for Interprofessional Learning Scale (RIPES) was used for data collection. The content validity of the questionnaire was confirmed by an expert panel (*n* = 4) and the content validity ratio (CVR) calculation. Its face validity was evaluated by administering it to 26 dental students and calculating CVR. The effects of demographic variables (gender, marital status, academic year, previous history of filling out the questionnaire, and participation in IPE courses) on the attitude of students were analyzed by a linear regression model. The final score of students was then calculated.

**Results:**

Demographic variables had a significant effect on the total score (*P* < 0.05), and females, singles, and senior students acquired a higher score. The mean score was also significantly higher in those with previous experience filling out a similar questionnaire and participating in IPE courses (*P* < 0.05). The mean score of students’ readiness for IPE was 70.19 ± 14.29%, and the mean acquired score of 88.1% was higher than the optimal threshold of 60.

**Conclusion:**

The study’s findings underscore the positive attitude of dental students towards IPE, providing strong support for including and implementing this educational model in the dental curriculum.

**Supplementary Information:**

The online version contains supplementary material available at 10.1186/s12909-025-07505-z.

## Introduction

Health science education is a foundation for many social, cultural, and economic constructions in any community, and the promotion of public health and prosperity is among its most favorable outcomes [1]. In contemporary education, knowledge is categorized within the professionalism construct. As a result, the goal of academic education has shifted towards knowledge acquisition of subject-specific content. Consequently, it diminishes the importance and breadth of its overall scope. Considering interprofessional relations, it is imperative to balance academic education, avoiding extremes of pure professionalism and generalization [2]. Since no single profession can address all the healthcare needs of patients, different healthcare professionals need to collaborate as a team to serve them. It is essential to train our healthcare workforce to learn the techniques of interprofessional collaboration to overcome challenges they might face later during patient care [3].

Unfortunately, perceptions regarding interprofessional education among Iranian health professionals are very limited [4]. Oral diseases, encompassing various risk factors such as diet, hygiene, stress, nutrition, tobacco, alcohol, and trauma, share commonalities with numerous chronic conditions. Therefore, fostering collaboration with other healthcare professionals, guided by the common risk factor approach, is immensely beneficial and strongly recommended [4]. The fundamental principle of this approach underscores the importance of directing efforts toward modifying a select number of factors that influence a broad spectrum of diseases. Efficient teamwork can improve the quality of clinical care and outcomes and increase patient satisfaction. Thus, teaching teamwork skills has gained growing popularity [5]. Accordingly, universities are searching for the most efficient educational approaches to teamwork. The World Health Organization has also emphasized expanding teamwork instructions, especially in community medicine, and benefiting from the problem-solving approach in medical education. It also asked for specific attention to interprofessional education (IPE) as a novel approach in teaching healthcare fields [6].

According to Britain’s Center for the Advancement of Interprofessional Education, a leading organization in IPE, IPE occurs when professionals of two or more fields learn from and about each other to improve the quality of offered services by increasing their collaboration [7]. This definition has three key points: (I) education is defined by learning events, (II) learning from, with, and about each other is a prerequisite for active learning, and (III) the primary goal of this type of education is to increase the collaboration and improve the quality of patient care [8]. IPE was developed to train students from different healthcare fields to learn from and about each other and improve their interprofessional collaboration [9, 10]. In IPE, students of varying healthcare fields should be actively involved in teamwork with other healthcare professionals early in their education to acquire the required skills for successful teamwork [11]. Although IPE is included in professional healthcare programs at the postgraduate level, the students need to master interprofessional competencies for successful implementation early in their educational period [12]. Some researchers believe that IPE should be started during undergraduate programs to prevent the development of a negative attitude towards other professions, which would be hard to eliminate [13]. However, some others believe that learners should first perceive their professional role to have an acceptable performance as a team member. Thus, they recommend the inclusion of IPE in the final years of education [14].

The main goals of IPE include changing the negative attitude of healthcare workers towards each other, improving the self-esteem of healthcare workers and enhancing their communication with each other, improving the teamwork skills of the personnel, confronting problems that exceed the capacity of a professional worker, the elevation of job satisfaction and reduction of stress, training a flexible workforce, and merging expertise-centered care with a holistic approach [15]. Developing optimal communication skills is the key to improving teamwork. Students’ interest in learning teamwork skills also plays a fundamental role in successfully implementing IPE [16]. Thus, this study aimed to assess the attitude of Iranian dental students regarding IPE.

## Methods and materials

This cross-sectional study was conducted on 235 third-, fourth–, fifth—, and sixth-year dental students at Tehran University of Medical Sciences in 2020–2021. Publication was delayed due to administrative and COVID-19-related disruptions. The study protocol was approved by the university’s ethics committee (IR.TUMS.DENTISTRY.REC.1400.022).

### Sample size

Using multiple regression power analysis of PASS 11, the minimum sample size was calculated to be 230 students, assuming alpha = 0.05, beta = 0.2, number of independent variables = 5, and R2 = 0.05.

### Data collection

The Readiness for Interprofessional Learning Scale (RIPLS) was used for data collection. It was selected for its wide use, reliability, and prior validation in similar Iranian contexts. In the present study, which includes 19 questions with 5-point Likert scale answer choices (agree to disagree). It has three subscales: teamwork and collaboration, professional identity, and roles and responsibilities. It reportedly has high content validity with a Cronbach’s alpha = 0.9(17). The validity and reliability of the Persian version of this questionnaire have also been previously confirmed by Amini et al., reporting a Cronbach’s alpha of 0.85 for teamwork and collaboration, 0.86 for professional identity, and 0.92 for roles and responsibilities (18). We used a validated questionnaire by Amini et al.(18) Amini and her team translated the questionnaire into Farsi, and its reliability and validity were assessed at the School of Paramedicine, Tehran University of Medical Sciences. In the present study, a forward-backward translation process was used to ensure linguistic accuracy. After ensuring the accuracy of translation, the validity of the questionnaire was evaluated.

Students were encouraged to provide qualitative feedback on clarity and simplicity during face validity.

### Content validity

Four experts in medical education (two experts in medical education, a vice chancellor of education of the Dental School of Tehran University of Medical Sciences, and a skillful epidemiologist) were asked to rate the questionnaire’s questions regarding necessity and relevance to the topic. Accordingly, the content validity ratio (CVR) was calculated for each question. The Lawshe’s table was then used to assess the suitability of each question. According to this table, CVR values greater than critical CVR are suitable, and the respective questions do not need to be omitted. The highest CVR would be 1 [17].

We used a conservative CVR threshold of 0.99 due to the limited number of experts (*n* = 4), consistent with Lawshe’s recommendations.

### Face validity

Twenty-six dental students were randomly selected and asked to assess the questionnaire’s questions in simplicity and clarity. Accordingly, the CVR was calculated for each question.

Finally, the final version of the questionnaire was designed online, and its link was sent to 235 third—, fourth-—, fifth-—, and sixth-year dental students of Tehran University of Medical Sciences in 2020–2021. Publication was delayed due to administrative and COVID-19-related disruptions. The students were asked to fill out the questionnaire anonymously and assured of the confidentiality of their information. Since the final version of the questionnaire had 19 questions, and four scores were allocated to each correct answer, the total score could range from 0 to 76.

### Statistical analysis

Data were analyzed using SPSS version 24 (SPSS Inc., IL, USA). The scores of each question and students’ total scores were calculated and reported. The linear regression model was applied to analyze the effect of different variables on the questionnaire score. The level of statistical significance was set at 0.05.

## Results

Students were encouraged to provide qualitative feedback on clarity and simplicity during face validity.

### Content validity

The calculated CVR for the necessity and relevance of each question are shown in Table 1. The critical CVR was found to be 0.99 in the present study, according to Lawshe’s Table [20]. Thus, question 18 was omitted since its CVR for both necessity and relevance was 0.5. Since only 1 question was omitted, the optimal content validity of the questionnaire was confirmed. Also, some other changes were applied to the text according to the opinion of the experts. Accordingly, question 7 was modified as two questions, as follows:

**Table 1:** Calculated CVR for the necessity and relevance of each question for assessment of content validity

Number	Question	CVR for content validity	
		Necessity	Relevance
1	Learning with other students will help me become a more effective member of a healthcare team	1	1
2	Patients would ultimately benefit if healthcare students worked together to solve patients’ problems.	1	1
3	Shared learning with other healthcare students will increase my ability to understand clinical problems.	1	1
4	Communication skills should be learned with other healthcare students.	1	1
5	Team-working skills are essential for all healthcare students to learn.	1	1
6	Shared learning will help me to understand my own limitations.	1	1
7	Learning with healthcare students before qualification would improve relationships after qualification.	1	1
8	Shared learning will help me to think positively about other professionals	1	1
9	For small-group learning to work, students need to trust and respect each other	1	1
10	I do not want to waste my time learning with other healthcare students	1	1
11	It is not necessary for undergraduate healthcare students to learn together	1	1
12	Clinical problem-solving skills can only be learned from my department	1	1
13	Shared learning with other healthcare students with help me to communicate better with patients and other professionals.	1	1
14	I would welcome the opportunity to work on small-group projects with other healthcare students.	1	1
15	I would welcome the opportunity to work with other students and healthcare workers in lectures, educational programs, and workshops	1	1
16	Shared learning with help to clarify the nature of patient problems	1	1
17	Shared learning before and after qualification would help me become a better team worker.	1	1
18	I am not sure what my professional role will be.	0.5	0.5
19	I have to acquire much more knowledge and skills than other healthcare students.	1	1

Original form of question 7: Learning with healthcare students before qualification would improve relationships after qualification.

Question 7a: Learning with healthcare students before acquiring professional skills would improve relationships and collaboration with others after acquiring professional skills.

Question 7b: Learning with healthcare students after acquiring professional skills would improve relationships and collaboration.

Questions 10 and 22 were checking questions to assess the accuracy of the responses and, therefore, should not be asked consecutively. Thus, question 11 was moved to the location of question 18.

We used a conservative CVR threshold of 0.99 due to the limited number of experts (*n* = 4), consistent with Lawshe’s recommendations.

### Face validity

Table 2 presents the calculated CVR for the clarity and simplicity of each question for the assessment of face validity. The critical CVR was found to be 0.37 in the present study, according to Lawshe’s Table [17]. Thus, question 7 (since it scored low in both clarity and simplicity) and question 11 (since it scored low in simplicity) were found to be unsuitable and were modified as follows:

**Table 2:** Calculated CVR for the clarity and simplicity of each question for assessment of face validity

Number	Question	CVR for face validity	
		Clarity	Simplicity
1	Learning with other students will help me become a more effective member of a healthcare team	0.53	0.46
2	Patients would ultimately benefit if healthcare students worked together to solve patients’ problems.	0.53	0.46
3	Shared learning with other healthcare students will increase my ability to understand clinical problems.	0.69	0.39
4	Communication skills should be learned with other healthcare students.	0.53	0.38
5	Team-working skills are essential for all healthcare students to learn.	0.84	0.53
6	Shared learning will help me to understand my own limitations.	0.61	0.69
7	Learning with healthcare students before acquiring professional skills would improve relationships and collaboration with others after acquiring professional skills.	0.23	0.07
8	Shared learning will help me to think positively about other professionals.	0.76	0.61
9	For small-group learning to work, students need to trust and respect each other.	0.46	0.39
10	I do not want to waste my time learning with other healthcare students.	0.46	0.53
11	Learning with healthcare students after acquiring professional skills would improve relationships and collaboration with others.	0.69	0.30
12	Clinical problem-solving skills can only be learned from my own department.	0.46	0.39
13	Shared learning with other healthcare students with help me to communicate better with patients and other professionals.	0.69	0.61
14	I would welcome the opportunity to work on small-group projects with other healthcare students.	0.69	0.61
15	I would welcome the opportunity to work with other students and healthcare workers in lectures, educational programs, and workshops.	0.69	0.53
16	Shared learning with help to clarity the nature of patient problems.	0.61	0.39
17	Shared learning before and after qualification would help me become a better team worker.	0.69	0.61
18	It is not necessary for undergraduate healthcare students to learn together.	0.69	0.69
19	I have to acquire much more knowledge and skills than other healthcare students.	0.46	0.61

Original form of question 7: Learning with healthcare students before acquiring professional skills would improve relationships and collaboration with others after acquiring professional skills.

A modified form of question 7: Learning with healthcare students, such as nursing, dental, and nutrition students, before acquiring professional skills would improve relationships and collaboration with others through teamwork practice.

Original form of question 11: Learning with healthcare students after acquiring professional skills would improve relationships and collaboration with others.

Modified form of question 11: Learning with healthcare students, such as nursing, dental, and nutrition students, after acquiring professional skills would improve relationships and collaboration with others through teamwork practice.

Thus, after modification of the abovementioned two questions, the face validity of the questionnaire was optimized.

### Descriptive findings

Table 3 shows the measures of central dispersion for the total score, gender, marital status, academic year, previous history of filling out the questionnaire, and previous experience with IPE. All confounding factors, including previous exposure to IPE and prior completion of the questionnaire, were adjusted for using regression analysis. The mean score of students was 53.3 ± 10.86 (range 15 to 76). The scores were standardized (calculated out of 100) for easier comparison with other studies. The mean score of females was higher than that of males. The mean score of singles was also higher than that of married individuals. The mean score of students increased by their academic year. Most participants (97.4%) had not completed this questionnaire before. Also, most participants (97.9%) had no experience in IPE.

**Table 3:** Measures of central dispersion for the total score, gender, marital status, academic year, previous history of filling out the questionnaire, and previous experience in IPE

		Number	Mean	Std. deviation	Minimum	Maximum
	Score	235	53.344	10.86	15.00	76.00
	Percentage	235	70.190	14.29	19.74	100.00
Gender	Female	125	71.252	14.67	19.74	100.00
	Male	109	68.964	13.88	22.37	100.00
Marital status	Single	175	71.812	13.36	22.37	100.00
	Married	60	65.460	15.92	19.74	100.00
Academic year	3rd year	41	68.581	17.09	23.68	92.11
	4th year	53	68.396	17.10	19.74	93.42
	5th year	42	71.084	14.34	21.05	93.42
	6th year	99	71.438	11.07	48.68	100.00
Filling out the questionnaire before	Yes	6	82.894	14.38	57.89	100.00
	No	229	69.857	14.17	19.74	100.00
Previous experience in IPE	Yes	5	67.857	12.22	67.11	100.00
	No	230	69.819	14.13	19.74	100.00

As shown, all variables had a significant effect on the attitude of students towards IPE (*P* < 0.05)) Table 4. (Females acquired a significantly higher score by an average of 3.07 units than males. Singles had a significantly higher score than married individuals by 5.108 units. Per each one-year increase in the academic year, the mean score of students increased by an average of 1.387 points. The mean score of those who had previously filled out the questionnaire was significantly higher than those without such an experience by 8.592 points. Those with previous knowledge of IPE had a significantly higher score than those without such experience by an average of 11.571 points.

**Table 4:** Results of linear regression analysis regarding the effects of different variables on the attitude of students towards IPE

Model	Unstandardized coefficients		STANDARDIZED COEFFICIENTS		
	B	Std. error	Beta	t	P-value
Gender	-3.037	1.393	-0.139	-2.180	0.030
marital Status	-5.108	1.581	-0.205	-3.231	0.001
Academic year	1.387	0.606	0.147	2.288	0.023
Previous experience in filling out the questionnaire	8.592	4.362	0.125	1.970	0.049
Previous experience in IPE	11.571	4.973	0.154	2.414	0.017

Lower IPE scores among male and married students may reflect cultural or structural barriers such as professional hierarchies and less exposure to collaborative practice.

## Discussion

This study assessed the attitude of Iranian dental students towards IPE. The results showed a mean attitude score of 70.19 ± 14.29%. The score acquired by 88.1% of students was above the optimal threshold of 60%, as reported by previous studies [18]Thus, it appears that most students had a positive attitude towards IPE, which agrees with the results of Sharifian et al. [18], who evaluated medical and midwifery students at the Iran University of Medical Sciences. The present results highlighted the optimal perception of dental students towards the role and significance of other healthcare workers in patient care and understanding the need for IPE.

The present results showed that all the tested variables significantly affected students’ attitudes towards IPE. Female students had a more positive attitude towards IPE than male students, which may be because females often have a higher spirit for cooperation and collaboration with others. Wilhemsson et al. [19] indicated that female students had a more positive attitude towards teamwork than male students, which aligned with the present findings. Single students had a more positive attitude towards IPE than married individuals. Also, students’ attitudes improved with an increase in their academic year of education. This finding may be because students at higher educational levels acquire a more comprehensive understanding of their roles and shortcomings in patient care and better feel the need for collaboration with other healthcare workers.

Dental students who had filled out this questionnaire had a more positive attitude towards IPE than those who had not filled it out before, which may be because those who had previously filled out this questionnaire gained more information, improving their attitude. Also, those with previous experience in IPE had a more positive attitude than others, which can be attributed to the fact that expertise in IPE improves students’ attitudes in this respect. McGregor et al. [20] indicated that students had a more positive attitude towards IPE after completing the course compared to their attitude at the onset of the course. Pogge et al. [21] demonstrated a higher knowledge level and a more positive attitude towards IPE in those who participated in the course. In contrast, Vahabi et al. [22] showed that demographic factors did not affect students’ attitudes toward IPE. Differences between their results and other studies, including the present investigation, may be due to different study populations. The item “Clinical problem-solving skills can only be learned from my department” had the highest frequency of disagreements since almost half of the students disagreed with this statement. This finding may be because this statement emphasizes the necessity of IPE only with the personnel of the same organization and denies IPE with members of other universities and organizations.

Previous studies showed statistically significant improvement in professional identity among pre-licensure learners, meaning that there was a readiness for shared expertise with other students through team-based approaches to learning as opposed to the typical discipline-based approach to learning [23]. A recent systematic review revealed that IPE was effective in improving attitudes towards pre-licensure learners and professionals of other disciplines, as well as increasing the value placed on a team-based approach for improving patient care [9]. Gomez et al. [24] evaluated an interprofessional education program in pediatric dentistry, medicine, and nursing. They concluded that this program provided important perspectives on oral health for medical and nursing students, which they did not receive elsewhere in their graduate education. The present study highlighted that students with previous IPE exposure scored significantly higher in readiness. This aligns with the findings of Fatahzadeh et al., who implemented an oral-medicine-centered interprofessional program that significantly improved students’ collaborative attitudes and cross-disciplinary understanding. Their success supports the argument that contextual, discipline-specific IPE—like dental-medical integration—can meaningfully impact student readiness [25].

Recent studies have consistently demonstrated the value of integrating IPE into dental education, not only through in-person formats but also through adaptable virtual environments. Chavis et al. reported that both in-person and online interprofessional education significantly enhanced dental and dental hygiene students’ attitudes toward teamwork and collaboration. These findings underscore the versatility and relevance of IPE in modern educational contexts, especially post-pandemic, supporting our observation that students with prior IPE exposure demonstrated more positive attitudes [26].

Structural and cultural barriers within healthcare education, such as role ambiguity and limited exposure to other disciplines, are well-documented impediments to interprofessional collaboration. Hollaar et al. [27]emphasized the role of early educational interventions to mitigate these issues. Similarly, Farrukh et al. [28] pointed to the necessity of faculty readiness and simulation-based education in creating successful IPE environments. These observations are directly relevant to the Iranian context, where interprofessional experiences are limited and often informal. Our findings suggest that formal curricular inclusion, paired with faculty training, could bridge these gaps.

A broader view of IPE reveals its implications for patient care quality and safety. Guraya et al. [29] conducted an interventional study showing improved awareness of safety and collaborative practice among healthcare students following an IPE workshop. This reinforces our argument that enhancing student readiness through IPE is not merely a pedagogical goal but a vital strategy to improve future clinical performance and patient outcomes.

The results of this study encourage and support educational institutions and universities to include IPE in their dental curriculums. Interprofessional education has been proposed as an alternative to traditional methods among medical and healthcare students. Interprofessional education has positively affected the learning process, the quality of patient care, and the professional performance of students [30]. To be successful in an interprofessional educational design, changes beyond place and structure must be applied. Success comes with fundamental changes. Cultural differences, such as disciplinary stereotypes and communication barriers, must be resolved. Students should be taught to be team members and communicate with other healthcare professionals [31].

## Conclusion

This study showed that among the dental students at Tehran University of Medical Sciences, unmarried female students in the higher academic year who had experienced the previous interprofessional training course and those who had filled out this questionnaire before had a more positive attitude and view. Towards interprofessional training and considered this training to improve their spirit of cooperation. They believe that acquiring teamwork skills is necessary for all medical sciences students because it makes them realize their weaknesses, limitations, and abilities. All the evaluated variables significantly affected dental students’ attitudes toward IPE.

Limitations of this study include its cross-sectional design, single-site sampling, and reliance on self-reported data.

## Electronic supplementary material

Below is the link to the electronic supplementary material.

Supplementary Material 1

## Data Availability

No datasets were generated or analysed during the current study.

## Abbreviations

IPE: Interprofessional education
RIPES: Readiness for Interprofessional Learning Scale
CVR: Content validity ratio
